# Child Health Partnerships: a review of program characteristics, outcomes and their relationship

**DOI:** 10.1186/1472-6963-10-172

**Published:** 2010-06-17

**Authors:** Kapila Jayaratne, Margaret Kelaher, David Dunt

**Affiliations:** 1 Centre for Health Policy, Programs and Economics, School of Population Health, The University of Melbourne, Level 4, 207 Bouverie Street, Carlton, Victoria 3010, Australia; 2 Child Health Unit, Family Health Bureau, Ministry of Healthcare and Nutrition, 231 De Saram Place, Colombo 10, Sri Lanka

## Abstract

**Background:**

Novel approaches are increasingly employed to address the social determinants of health of children world-wide. Such approaches have included complex social programs involving multiple stakeholders from different sectors jointly working together (hereafter Child Health Partnerships). Previous reviews have questioned whether these programs have led to significant improvements in child health and related outcomes. We aim to provide definitive answers to this question as well as identifying the characteristics of successful partnerships.

**Methods:**

A comprehensive literature search identified 11 major Child Health Partnerships in four comparable developed countries. A critical review is focused on various aspects of these including their target groups, program mechanics and outcomes.

**Results and Conclusions:**

There was evidence of success in several major areas from the formation of effective joint operations of partners in different partnership models to improvement in both child wellbeing and parenting. There is emerging evidence that Child Health Partnerships are cost-effective. Population characteristics and local contexts need to be taken into account in the introduction and implementation of these programs.

## Background

Recognition of the need to address the social determinants of health has led to innovative mechanisms to improve the quality of outcomes in child health being developed. The development of strategic partnerships between service providers from different sectors and the community is central to many of these innovations. The characteristics of these partnerships are well captured in the following terms. "Partnership is a dynamic relationship among diverse actors, based on mutually agreed objectives, pursued through a shared understanding of the most rational division of labour based on the respective comparative advantages of each partner. Partnership encompasses mutual influence, with a careful balance between synergy and respective autonomy, which incorporates mutual respect, equal participation in decision-making, mutual accountability, and transparency" [1] (p.216).

The extent and closeness of partnerships between service providers range on a continuum from networking, co-ordination, co-operation to collaboration [2]. Networking involves the exchange of information for mutual benefit. Co-ordination involves information exchange for mutual benefit and altering activities for a common purpose and requires more time, higher levels of trust but little or no access of each other's domain. Co-operation includes many of the aspects of co-ordination - information exchange for mutual benefit, altering activities for a common purpose and sharing resources for mutual benefits and a common purpose. It also entails large amount of time, high levels of trust, significant access to each other's domains and complex organizational processes and (written) agreements [2-4]. Collaboration is comprised of the activities of networking, co-operation and co-ordination listed above, but also involves improving the capacity of the other partner for mutual benefit and a common purpose [2]. Some commentators question whether it is important to make such subtle distinctions between these terms or rather to assume they are broadly similar or identical and capable of being interchanged [5].

Initiatives aimed at improving child health, including Child Health Partnerships must address the large number of social and economic factors affecting health and wellbeing [6]. Child Health Partnerships by their nature are particularly suited to address these complex factors [7] and to achieve the various outcomes that cross the early childhood sector and have as a result become increasingly adopted [5]. With no preferred Child Health Partnerships being identified, restructuring of systems across programs and agencies is occurring world-wide. Concepts are still evolving and may have different manifestations depending on the needs and the context of the each program [8] and the different priorities of the partnering agencies involved.

Notwithstanding these arguments in favour of joint work [5], working in partnerships has frequently been assumed to be more difficult and expensive [9,10]. In even quite recent reviews of service integration in early childhood initiatives, Valentine et al [10] reported mixed results with a majority of programs not showing the ability to achieve better impacts on child outcomes. Janie [11] also appraised the development and delivery of effective partnerships for children (including reviews of them) and was unable to report substantial robust evidence of definitive impacts. Dowling et al [5] found limited success in health and social care partnerships generally (ie not specifically in relation to the childhood area). Valentine et al [10] makes the point that it is necessary to compare integrated versus fragmented services to attribute positive outcomes to the workings of a partnership. However such evidence is rare in literature.

With this background, it is essential therefore to reflect on the effectiveness of Child Health Partnerships initiatives and ensure that they constitute an appropriate investment. In this paper we review key child health programs which were based on partnerships, some of which have only been reported very recently. We develop a typology of different types of partnership arrangements and examine whether the type of partnership has any impact on their success.

The framework outlined by Dowling [5] has been used to conceptualize the success of Child Health Partnerships in terms of two key dimensions.

The implementation of partnerships:

• Was the partnership formation successful?

• Was there quality in continuation of partnership?

• How did the partnerships contribute to service delivery and/or service utilization?

The improvements in the health status or related parameters of users:

• Was there positive early childhood development in major domains?

• Were there improvements in parents and/or families?

Despite much writing on the conceptual and theoretical framework on the operation of Child Health Partnership, this does not in itself provide critical review of their outcomes both organizationally and in terms of child health and wellbeing outcomes. This review aims to redress this.

## Methods

It is necessary first to define Child Health Partnership. A consensus definition for Child Health Partnership is hard to find in the research literature. We constructed a working definition based on the currently available descriptions.

### Definition: Child Health Partnership

A comprehensive organisational framework made up of two or more local partnering agencies working towards a common objective of ensuring the physical and social development of young children.

We conducted a literature search to identify specific child health programs targeting early childhood care and development. We included only systematic government initiatives which involved multiple stakeholders in partnership building and programs sustained for more than three years. We restricted the review to programs that operated in the past two decades (1989 - 2009).

The search included general databases; PubMed, Web of Science (ISI), PsycINFO (CSA), MEDLINE (ISI), Google and University of Melbourne Library Catalogue. (Since MEDLINE (ISI) includes a wider coverage of multidisciplinary biomedical literature in line with our topic, CINAHL database which covers nursing or allied health literature was not included in the search). In exploring the databases, we used multiple, alternative terms denoting community-based early child health programs combined with Boolean operators (OR) for the title. The words included a range of synonyms and derivative terms signifying 'early year health programs' and 'partnership'. Then such separate searches were combined using 'AND' to export the yielding citations to EndNote, the reference manager software. We screened pooled EndNote citations to select abstracts of interest to retrieve full articles.

Since this exercise did not yield significant number of articles, we perused bibliographies of the selected articles to locate further publications. Additional references were searched using PubMed and Google to obtain related articles.

Characteristics of the selected child health initiatives were mapped in tabular format focusing on target groups, partnering agencies, highlights of the program model and main activities (Additional file 1), and evaluation design and evaluation outcomes (Additional file 2).

#### Inclusion criteria

1. Systematic programs launched either at national or state/county level

2. Involved more than two stakeholders in partnership building or service integration

3. Partnerships are based at local areas

4. Sustained for a sufficient length of time (at least two years)

5. Operated in the past two decades (1989 - 2009)

#### Exclusion criteria

1. Programs aimed at improving parameters other than early childhood outcomes

2. Programs focused entirely on one particular parameter of early childhood development

## Results

Our search identified 11 child healthcare partnership programs in four countries. The main features of these programs are set out in Additional file 1.

(See: Additional file 1 Child Health Partnerships Projects - Program characteristics )

### Partnership programs in child health

Child Health Partnership programs exist in the United Kingdom (UK), United States (USA), Canada and Australia. These are *Sure Start *(UK), *Every Child Matters *(UK), *First 5 California *[12], *Early Head Start *[12], *Healthy Child Manitoba *(Canada) and *Toronto First Duty *(Canada). Australia has also initiated child health intervention programs working collaboratively in every state and territory [10]. These include *Families First *(New South Wales), *Stronger Families and Communities *(Commonwealth), *Every Chance for Every Child *(South Australia/Victoria) and *Best Start *(Victoria).

### Target group

The Child Health Partnerships targeted a wide spectrum of children. Many included all children in the area though with varying ages (eg. *Sure Start*- before birth to 4 years old, *Every Child Matters*- from conception through to age 14, *First 5 California*- from prenatal to five years of age). A majority of programs included not only children but also their families or care givers and communities.

*Sure Start, Early Head Start *and *Best Start *focused on children and families living in disadvantaged areas. In such area-based initiatives, all families living within the targeted areas were included in the interventions. In *First 5 California*, families included extended families and other primary caregivers. *Families First *and *Best Start *widened their services to include even communities. When *Sure Start *evolved to become *Every Child Matters *and UK-wide, all children up to 14 years of age and their families in the area were included. There was though a particular focus on the most disadvantaged. In *Stronger Families and Communities*, target groups varied across the whole program area serviced by the program, in one part a whole 'at risk community' and in another sub-groups only within a community. *Every Chance for Every Child *(South Australia/Victoria) focused on vulnerable children, young people and families.

### Partnering agencies

The composition of the partnership varied from program to program and area to area. A common partnership model was cross-agency collaboration of non-governmental and community services with health as the core service element or the lead agency. An exception was *Every Chance for Every Child *which was a joint partnership of human and legal services. Several other statutory services (education/local councils/human services) participated to varying extents. In *Sure Start*, an alliance of early education, childcare, health and family support services was established. This included mainstream agencies concerned with provision of services to children and families.

The education sector had prominent roles in *Every Child Matters*, *First 5 California*, *Early Head Start*, *Healthy Child Manitoba*, and *Toronto First Duty*. Only two of the Australian programs *Best Start *and *Families First*, had such an involvement and a third, *Families First *(New South Wales) being a collaborative effort involving health, community, education and human services.

Private and volunteer groups together with non-governmental organizations (NGOs) formed alliances in several programs. In *Stronger Families and Communities*, NGOs were both lead agency and 'facilitating partner' for the receipt and allocation of funding. They were to involve other stakeholders in the community [13]. Other programs with NGOs as contributing partners were *Early Head Start*, *Healthy Child Manitoba *and *Families First*. Family involvement was notable in a majority of programs eg. *Sure Start, Every Child Matters, Stronger Families and Communities *and *Best Start*.

### Program model

Many programs contemplated a reorganization of existing services within existing resources and the development of regional linkages between partnering agencies eg. *Best Start, Stronger Families and Communities *and *Families First *[6]. While developing partnerships across the early child development sector, *Best Start *aimed also to improve service co-operation and co-ordination across the whole sector not just between *Best Start *partners. New services were not introduced nor existing services expanded. In general, its strategies were based on social marketing, cross-service promotion and co-ordination, reminder systems and play group formation. *Sure Start *employed partnership models across different service delivery systems to achieve better results [14].

*Stronger Families and Communities *used the 'Facilitating Partner Model' with an NGO in the lead role, as a vehicle for distributing government funding. As set out in Additional file 1, there were four strands: *Communities for Children *(CfC), *Invest to Grow *(ItG), *Local Answers *(LA) and *Choice and Flexibility in Child Care*, each having different aims [13]. Only 78 of the 191 sites focused on early intervention in early childhood.

Most initiatives used outreach services - some others used centre-based service delivery eg. *Sure Start (Every Child Matters)*, *Early Head Start *and *Toronto First Duty. Early Head Start *used three specific program modes- centre-based, home-based and mixed approaches. For example, home-based service delivery involved weekly home visits and at least two group socializations per month for each family [15].

Children's Centres (*Every Child Matters*) evolved from earlier *Sure Start *Children's Centres. They were conceived as one-stop shops, providing high-quality integrated childhood services in co-located sites to communities. They had as their overall goal to improve child outcomes. They varied service delivery according to the level of local disadvantage. Their funds derived from pooled funding streams that were previously allocated for early education, childcare and other family focused services in disadvantaged areas.

*Every Chance for Every Child *deviated from other programs by focusing on child abuse and neglect.

### Activities

Almost all programs show varying service components. In these programs activities were developed according to local needs and therefore, many of them did not have a prescribed set of activities. In some the activities were different even within the program based on the locality. The service delivery was modelled mainly on family support, domiciliary services and early learning opportunities. The range of services addressed health issues from conception up to teen age. They started with antenatal support (*Every Chance for Every Child*), breastfeeding promotion (*Best Start*), early learning facilitation (*Sure Start*, *Toronto First Duty *and *Every Chance for Every Child*), school education (*First 5 California*) and services for young people (*Sure Start *and *Healthy Child Manitoba*). However, the integrity of the intervention -the "dose" delivered at an optimal level across all sites [16] in a similar fashion- is not readily apparent.

Facilitation of early learning was a core activity in almost all programs. Several programs have paid attention to nutritional support e.g. *Every Child Matters, Healthy Child Manitoba, Stronger Families and Communities *and *Best Start*. Apart from catering for individual children, many programs have widened the spectrum of services to reach families and to end up in community capacity building (*Every Chance for Every Child*). In *Every Child Matters*, a notable feature is the provision of distinct services to families to cater for different needs, both between different families, in different locations and across time in the same family. Even though the service delivery was networked through a Children's Centre, the strategies used outreach and home visiting to improve access to services for families who are unlikely to visit a centre.

Provision of family support can be identified in several ways; financial support, provision of basic family needs, parenting support and different modes of child care services. Several programs have provided financial assistance in different ways e.g. *Every Child Matters *- tax credits to cover childcare costs, *First 5 California *- health insurance enrolment assistance and provision of basic family needs (food, clothing and housing). In provision of child care services, different programs have used different methods; traditional child care services, child minding, out of school childcare and assistance in childcare costs.

Only three programs (*Sure Start *(UK), *Every Child Matters *(UK) and *First 5 California *(USA)) have launched services for children with special needs. None of the Australian initiatives had defined activities focused on them though they have targeted vulnerable/disadvantaged children. Needs of the young people have been addressed by *Sure Start *and *Healthy Child Manitoba*.

*Every Chance for Every Child*, with its specific focus, has employed community-based child and family services for abused children and children in care.

### Evaluation designs

Additional file 2 summarizes available information on evaluation designs and evaluation outcomes for the seven programs that were evaluated.

(See: Additional file 2 Child Health Partnerships Projects -Evaluation designs and outcomes)

Many programs used robust evaluation study designs usually quasi-experimental trials but also randomized trials. Both *Sure Start *programs, *Toronto First Duty *and *Best Start *used quasi-experimental study designs and *Early Head Start*, a randomized trial. *Families First *and *Stronger Families and Communities *used triangulated methodologies with both quantitative and qualitative elements.

Quasi-experimental evaluations were conducted in *Sure Start *and *Best Start *programs at area-level. An area-based comparator was present for both *Sure Start *(compared with whole of England) and *Best Start *(compared with non-*Best Start *program Sites).

A variety of measures were used to assess aspects of program delivery. Partnership formation was assessed by all of the seven evaluated programs excepting *Early Head Start*. A number of programs undertook process evaluations tailor-made to distinct program characteristics of the projects under review. *Best Start *and *Stronger Families and Communities *assessed service cooperation and co-ordination. *Best Start *used a purpose-designed tool to assess the number and type of service co-operation activities. *Stronger Families and Communities *used several inventories to compute a Helpfulness score, Day-to-day co-ordination indicator and Effective partnerships indicator in the evaluation of inter-agency activities. To assess the extent of partnership, *Sure Start *focused on management and co-ordination with a 'joined-up-ness scale' while *Toronto First Duty *used indicators of change to benchmark the different phases of partnering. In *Families First*, the extent of success of service integration was evaluated using a network density matrix to assess the strength of the network linkages between partners.

Child health and parenting outcomes measures were used in the six programs. Both *Sure Start *programs used computer-assisted interviews/parent reports with specific scales for child development, parenting and family functioning. In *Early Head Start*, child outcomes were assessed with child development scales. Parenting outcomes were assessed with Home Observation for Measurement of the Environment (HOME) tool. The Early Development Instrument (EDI) assessed child health outcomes in *Toronto First Duty*. Statutory data sources e.g. immunization records were used in *Best Start *to measure some child and family outcomes. *Families First *has not reported its child health and parenting outcomes.

An economic evaluation was incorporated in the study designs of *Toronto First Duty, Stronger Families and Communities *and *Sure Start*. *Stronger Families and Communities *employed a cost-effectiveness study with development of cost-benefit matrices and reviews. *Sure Start *conducted a descriptive cost-effectiveness analysis. *Toronto First Duty *compared costs of integrated versus traditional service delivery.

### Outcomes

Positive outcomes of Child Health Partnerships were categorized either as forming partnerships or improvements in child health and parenting, wherever this was possible. Table 1 presents the proportion of all programs (denominator) with positive outcomes (numerator) for these to categories.

**Table 1:** Positive outcomes of child health partnerships

Positive outcomes (Improvements)	Proportion of programs	Indicated Programs
**Forming partnerships**		

Successful partnership formation among different stakeholders at local level	5/5	*Sure Start, Toronto First Duty, Families First, Stronger Families and Communities and Best Start*

Effective service cooperation and coordination in partnerships	4/5	*Sure Start, Toronto First Duty, Families First, Stronger Families and Communities and Best Start*

Wider community involvement	2/5	*Sure Start, Early Head Start, Toronto First Duty, Families First and Best Start*

Importance of both home and centre-based service provision	2/2	*Sure Start and Early Head Start*

Improved access to child health services for children and families	4/5	*Sure Start, Early Head Start, Toronto First Duty, Families First and Best Start*

Better service utilization by parents and families	4/5	*Sure Start, Early Head Start, Toronto First Duty, Families First and Best Start*

Sustainability of services	1/5	*Sure Start, Early Head Start, Toronto First Duty, Families First and Best Start*

Cost-effectiveness of integrated child health service delivery	3/4	*Sure Start, Early Head Start, Toronto First Duty and Stronger Families and Communities*

**Improvements in child health and parenting**		

Positive early childhood development in major domains: cognitive, social, emotional, language, literacy, nutrition and eating habits	3/5	*Sure Start (Children Centres), Early Head Start, Toronto First Duty, Stronger Families and Communities and Best Start*

Improved parenting outcomes	5/6	*Sure Start (Children Centres), Sure Start (SSLP), Early Head Start, Toronto First Duty, Stronger Families and Communities and Best Start*

Improved immunization uptake	2/2	*Sure Start (Children Centres) and Best Start*

All programs where the nature of the partnership was evaluated (Sure *Start, Early Head Start, Toronto First Duty, Families First, Stronger Families and Communities *and *Best Start*) were successful in these terms. Partnerships involved primary level services in several of these e.g. *Stronger Families and Communities *as well as *Best Start*. Only *Families First *reported partnership building limited to government bodies. There is evidence that service integration improved the quality of the child health programs e.g. *Toronto First Duty*.

There was improved service co-operation and coordination in four of the five programs studied. Effective partnerships were built with moderate levels of day-to-day coordination in *Stronger Families and Communities. Sure Start *and *Best Start *reported maturation of partnerships in all dimensions over time, somewhat delayed in the former. *Families First *demonstrated promising achievements in building partnership relationships and improving service access and provision. Wider community involvement was apparent only in two programs *Stronger Families and Communities *and *Best Start*. Trust among partnering agencies generated commitment from a wider community that had not been anticipated (*Stronger Families and Communities*) [13]. Both programs that trialled both home-based and centre-based activities reported positive outcomes (*Sure Start -Children Centres *and *Early Head Start*).

A proportion of programs that fostered linkages between stakeholders led to the formation of a 'holistic' service model catering for families. Access and service utilization by consumers typically improved. More families including more disadvantaged families were engaged e.g. *Best Start*. *Sure Start*, *Families First *and *Best Start *reported positive service uptake with important flow-on effects.

Most partnership programs that were subject to economic appraisal were found to be cost-effective (*Sure Start (SSLP), Toronto First Duty *and *Stronger Families and Communities*). *Stronger Families and Communities *sites with an alternate lead agency (NGO) reported enhanced short-term and long-term cost-benefits ratios in most sites [17]. Costs of the integrated and traditional program in *Toronto First Duty *were not substantially different. Only one program, *Stronger Families and Communities *however, reported sustainability in its partnership even after funding ceased.

Three of the five programs evaluated (*Sure Start-Children Centres, Early Head Start *and *Toronto First Duty*) reported a variety of child health development gains, including cognitive, language and social-emotional development of childhood (*Early Head Start *and *Toronto First Duty*). Parenting, outcomes improved in both *Sure Start *programs, *Early Head Start, Toronto First Duty *and *Stronger Families and Communities*. Better parenting (*Sure Start-Children Centres*) led to child social improvements. In *Stronger Families and Communities *partnership building among agencies at local level was deemed to lead to improved family and better community wellbeing.

## Discussion

The characteristics of the formation of partnerships as well as their outcomes can be considered under a number of themes below.

### Target group

In almost all programs, the program model and activities included not only the child but also their family or broader community. While the goal of many programs was to target the most disadvantaged children and families, universally available services were also provided. Such an approach sought to enhance equity and avoid social stigmatization. There was considerable variation between programs in targeting other vulnerable children such as those with special needs.

### Partnering agencies

The formation of partnerships varied depending on locality, whether service sectors were included and the level of engagement with their community. On most occasions, government bodies had taken up the ownership or key role. A consortium of local government bodies with different sectors and community stakeholders built the nucleus of partnerships in most cases. Health services predominantly took the lead role in a majority of partnerships. There are instances that other organizations e.g. NGOs could do the 'job' [13]. In several programs, mainstream services provided by local authorities and the national health system were supplemented.

### Program model

There was no common program model across partnerships and an understanding of the model underlying each Child Health Partnership is central to understanding their operation and achievement. Programs administered by partnerships also differed since services needed to be adjusted to the needs of the local community. All partnerships however used service integration to some extent in pursuing program goals.

### Activities

While on-the-ground services varied depending on the specific needs of the beneficiaries, program activities were launched around a common broad framework, activities were based on three overlapping levels of functions: system-level, organizational-level and client-level described by King and Meyer [18]. The majority of programs involved a mixture of early learning programs, home visits, outreach services, childcare services and support to parents and families. Programs targeting disadvantaged children and families should generally include domiciliary or outreach (door-step) services which improve access to services for families unlikely to visit centres for whatever reason.

### Evaluation designs

Evaluation provides a systematic reflection on the value of programs and their achievement. Many Child Health Partnerships employed high quality evaluation designs. Distinct methods included: quasi-experimental evaluations with either pre/post or longitudinal methods of data collection; qualitative and quantitative; area- and individual-based. Some employed both mixed quantitative and qualitative designs. The sceptical view that rigorous methodologies could not be employed in evaluation of complex social programs such as Child Health Partnerships was confounded [7].

Investment in early childhood development has previously been demonstrated to produce a high return on human capital formation [19,20]. Child Health Partnership programs are complex emergent programs and difficult to evaluate [21] nevertheless they have the potential to improve decision making around resource allocation. The four programs that undertook such appraisals demonstrated positive outcomes.

### Outcomes

Overall the evaluation of Child Health Partnership programs demonstrated positive outcomes. This was at variance to earlier reports that health partnerships [5] and more specifically Child Health Partnerships [10,11] had limited effect. The spectrum of positive outcomes ranged from the formation of successful partnerships to the achievement of enhanced child wellbeing assessed using a variety of measures especially in health, social and educational areas - Table 1 summarizes these. There was however variability of effect size which may be due to differences in program implementation, population characteristics as well as different evaluation designs [22]. The obvious variations in the cultural contexts and organization and structure of administrative sectors have to be taken into account. There was also a lack of useful information on processes and mechanisms of program activity in many programs.

*Partnership formation: *Five of seven programs with evaluation information reported successful partnership formation among local stakeholders. Many of the stakeholders to these programs were common - from local bodies/councils, Non-Government Organisations, families and community partners. Health-led partnerships were shown to be most successful in *Sure Start*.

Effective service cooperation and coordination in many programs demonstrated the 'healthiness' of the partnerships that were formed. Interestingly programs focused on evaluating partnership dynamics; *Families First*, *Stronger Families and Communities *and *Best Start*, reported a range of positive outcomes along the continuum of partnership arrangements.

Improvements in service provision and access to services were notable in most programs. They also had similar partnership composition, activity profile and better partnership formation.

Better service utilization by intended and current users (parents and families) was reported by the two *Sure Start *programs, *Toronto First Duty *and *Best Start*. These programs reported improved service provision and access which can be assumed to have led to this better utilization. Again these four programs shared similar partnership composition and program activities. They utilized existing services along with some further expansions.

*Child and parenting outcomes: Early Head Start, Every Child matters *and *Toronto First Duty *showed promising early childhood and parenting outcomes. *Toronto First Duty *demonstrated the ideal sequence of partnership formation - its maturation along a continuum of partnership, improving service delivery, better service utilization by recipients leading to positive parenting and child health outcomes. This is also true for *Every Child Matters*, if *Sure Start *was considered as its first phase. A common feature of these programs was their lengthy duration of implementation and high quality evaluation designs. Positive parenting outcomes were consistent with positive outcomes of children in all programs. This is not surprising since family environment is a key predictor of major domains of child wellbeing [23].

### Relationship of outcomes with program characteristics

It is challenging to relate outcome success to program characteristics given the variability of the programs. To achieve this we considered quality of evaluation design, program maturity/quality and better outcomes.

Targeting disadvantaged families, outreach service provision and partnerships among local level stakeholders were common to achieving better impacts. Attention to the processes of partnership building so as to achieve program maturity as successful partnership formation was a prerequisite for good program outcomes. Better child health outcomes were shown by programs that employed high quality evaluation designs. These were able to demonstrate that these outcome results were related to outreach service provision and targeting disadvantaged families. Better parenting outcomes were associated with improved early childhood development outcomes.

The positive child health and parenting outcomes of established *Sure Start *Children's Centres in contrast to *Sure Start *Local Programs emphasize the need for clearly focused and specified activities that is possible as programs mature [24].

Programs with higher quality evaluation designs may be associated with improved outcomes since presumably these designs are more able to demonstrate effects when they exist. It is also possible community interventions may show weak effects even with cluster randomized controlled trial evaluation designs as it is not possible to enforce identical program interventions in the intervention localities [16]. Not only are the interventions complex, so too are the communities [16]. This necessitates the use of mixed qualitative and quantitative methods in evaluating complex health interventions [25].

Success has not been demonstrated in some areas such as housing and school performance even by well conducted programs like *Families First *[6]. Slower progress in these areas may be associated with the difficulty of change in these areas or may reflect the fact that most partnerships were health-led.

### What do the results mean for the future of Child Health Partnership programs?

The positive results for the economic appraisals of these Child Health Partnership models [26] constitute a firm basis for their wider dissemination. Many of the programs in fact were based on existing services that did not require large increases in funding. This suggests that they may also be suitable in resource-poor settings.

Program characteristics change over time and across sites to cater for changing and different local needs and opportunities. Partnership building is the rate-limiting step in this. As noted, positive outcomes are more apparent in programs with longer program implementation time. An exemplar is the *Toronto First Duty*. *Every Child Matters *represents an evolution of the concept underlying *Sure Start *service delivery. The initial evaluation of *Sure Start *did not show positive effects on the most disadvantaged families [27] while in the evaluation of more established program (*Every Child Matters)*, positive outcomes were apparent [24]. This has been attributed to the improvements in service effectiveness with program maturation and longer exposure of the target groups to the services [28].

Positive outcomes with future programs will be more likely if the contextual factors that were important to building partnerships and programs were identified in their evaluations. This is because learning in relation to which contextual factors are important can better inform partnership building in the future [29].

Effective interventions in Child Health Partnership programs have the potential to address the consequences of early childhood development due to social inequalities [23]. There is also some evidence that many child health outcomes have been improved without a significantly increased family income [15]. This constitutes a further argument for the wider dissemination of these programs. Reducing disadvantage through these programs though is an intergenerational goal not an immediate one.

### Limitations

This review has concentrated on child health partnerships only in developed countries and findings may not be generalizable in other contexts. We report evaluation studies for seven programs each with their own design rather than a common design. While we draw conclusions with regard to grouped outcomes of programs, this should be interpreted cautiously due to variation in the programs and the quality of evidence that is assembled. Sophisticated evaluation designs covering all program dimensions took place in most but not all programs. In addition, the duration of program implementation varied with many programs still in the early phases of program maturity at the time of their evaluation.

It is difficult to propose a direct causal link between program characteristics (partnership formation) and outcomes. It is necessary to consider the quality and completeness of data, the robustness of evaluation design, scope of evaluation as well as the unique characteristics of particular programs [5].

## Conclusions

A majority of the Child Health Partnership initiatives which focused on joint-working with key stakeholders were successful, contrary to earlier reports. Take home messages given below recapitulates their main findings. Positive outcomes ranged from effective partnership formation along a continuum to achievement of enhanced child wellbeing and parenting outcomes. They have shown promising results in achieving short term outcomes and forming partnerships that were well if not fully developed. There is emerging evidence that child health service delivery in partnership model is cost-effective. It is difficult to determine what program model is best given the variability of program and local contexts. Viewed differently, the Child Health Partnerships under review argued that they were successful in meeting local objectives in bringing together several agencies with different capacities and objectives.

Take Home Messages

1. The particular characteristics of partnership programs should reflect local needs;

2. There was abundant evidence of successful partnership formations;

3. Programs with more maturity (longer program time) showed better child health and parenting outcomes;

4. Child health service delivery in partnership models is cost-effective;

5. Robust evaluation designs based on specific program models can be developed and employed in Child Health Partnerships;

6. Home and outreach service provision in Child Health Partnership are indispensable in addressing the needs of disadvantaged children and their families.

Good practice examples of Child Health Partnership programs based on service integration model will shape the future initiatives. Considering the difficulties in mapping outcome success, a commonly agreed framework in designing and evaluating child health partnership models is emphasized. Such efforts should begin with defining target groups, specific activities that should be incorporated in service provision, consideration on program quality and program maturity, agreement on programs impacts (more specifically child health and parenting outcomes) and a more generic evaluation design.

The need for assessment of the level of integration at policy, regional planning, program delivery and practice levels is essential to clarify implications of partnerships for practice [30]. We recommend an independent scoring system to measure the level of partnership formation for follow-up study. Long-term follow-up of the young child is necessary to assess the longer-term impacts of Child Health Partnership. Long term programs are also necessary to build and sustain partnerships [6].

## Competing interests

The authors declare that they have no competing interests.

## Authors' contributions

KJ, MK and DD contributed equally to conception, design and interpretation of data. KJ carried out the literature search, review and drafting the manuscript. MK and DD involved in critical revising of the manuscript.

All authors read and approved the manuscript.

## Pre-publication history

The pre-publication history for this paper can be accessed here:

http://www.biomedcentral.com/1472-6963/10/172/prepub

## Supplementary Material

Additional file 1 **Child Health Partnerships Projects - Characteristics of Programs **[31-40] Click here for file

Additional file 2 **Child Health Partnerships Projects -Evaluation designs and outcomes **[41-46] Click here for file
