# Supplementary material for: Child Health Partnerships: a review of program characteristics, outcomes and their relationship
Source: BMC Health Serv Res. 2010 Jun 17;10:172. doi: 10.1186/1472-6963-10-172 (PMC2908613; doi:10.1186/1472-6963-10-172)
Supplement: Additional file 1 — Child Health Partnerships Projects - Characteristics of Programs [31-40] [file 1472-6963-10-172-S1.DOC]

**Child Health Partnerships Projects - Characteristics of Programs**

| **Program** | **Target Group/s** | **Partnering agencies** | **Highlights of Program model** | **Activities** |
| --- | --- | --- | --- | --- |
| ***Sure Start: Local Programs***  (1999-2005 UK) [14] | Children (before birth - 4 years old) & families living in disadvantaged areas (Area-based) | Local bodies/councils  Health services  Social services  Education services  Families  Private & volunteer groups  NGOs  Communities [27] | Based on local programs (SSLP)  Existing services enhanced | Home visits  Outreach services  Support to families  Early learning programs  Support for good quality play  Childcare services  Services for children with special needs  Community healthcare [27] |
| ***Sure Start: Children Centres*** **(*Every Child Matters)***  (2005 UK) [31] | All children & families; children from conception through to age 14 years  Children with special educational needs and disabilities (up to age 16) | Local bodies/councils  Families  Private & Volunteer groups  Sure Start; Extended schools & childcare group joined together | Sure Start was transformed into children's centres (One-stop shop for child health services) after 2004 – 2006  Pooled budgets among partners | Home visits  Outreach services  Support to families  Parenting support  Early learning programs  Support for good quality play  Extended schools  Childcare/minding services  Services for children with special needs  Services for young people  Childcare costs –tax credits  Community healthcare |
| ***First 5 California***  (1998, USA)  [32] | All children from prenatal to five years of age;  Parents, relatives and primary caregivers of children | Schools  Health services  Childcare services  NGOs | Based on the California Children  and Families Act of 1998; comprehensive programs were established emphasizing community awareness, education, nurturing, child care, social services, health care and research | Provision of basic family needs  Parenting support  Parent/Caregiver education  Early learning programs  School readiness program  Childcare services  Immunization  Services for children with special needs  First smiles – dental education  Improved health access - health insurance enrolment assistance |
| ***Early Head Start***  (1994, USA)  [15] | Disadvantaged families  (low-income pregnant women and families with infants and  toddlers) | Volunteer/NGOs in community  Schools | Three program modes:  *Centre-based -*services through centre-based activities  *Home-based -*service provision by home visits  *Mixed approach -*centre-based / home-based or a mixture of them | Home visits  Parenting support  Early learning programs  Childcare services |
| **Program** | **Target Group/s** | **Partnering agencies** | **Highlights of Program model** | **Activities** |
| ***Healthy Child Manitoba***  (2000, Canada)  [33] | All children | Families  Health workers  Community partners  Schools  Early childhood workers | Utilization of existing and new service infrastructure.  Program planning and service delivery coordinated across sectors  Specific needs of each child and family considered for support | Home visits  Early learning programs  Parent-Child centred Approach  Triple P program  Financial support  Nutrition help in schools  Services for young people  Community healthcare/development |
| ***Toronto First Duty*** (2002, Canada).  [26, 34, 35] | All children | Community partners  Schools  Local bodies  NGOs | A single, accessible program with child care, kindergarten and family support services located in primary schools  Coordinated with early intervention and family health services | Parenting support  Outreach family support  Early learning programs  Childcare services in schools |
| ***Families First*** (New South Wales)  1998-2002 [36] | All children, families and communities | Area Health services  Community partners  Human Services Depts.  Dept of Ageing, Disability and Home Care  Dept of Community Services  Dept of Education and Training  Dept of Housing  Local bodies/councils  NGOs | Existing specialized health, community welfare, educational and other services utilized.  Regional linkages developed between services [37]  A model based on service integration and networking, community outreach and community development. | Home visits  Outreach family support  Childcare services  Community healthcare  Schools as community centres |
| ***Stronger Families and Communities*** (2004-2008, Australia)  [13] | All children (prenatal – 5 years) and families | NGOs  Community partners  Local bodies/councils  Families | Regional linkages between specialized health, community welfare, educational and other services  NGOs as lead agency and based on ‘facilitating partners’ concept.  Four strands: *Communities for Children* (CfC); *Invest to Grow* (ItG); *Local Answers* (LA); and *Choice and Flexibility in Child Care*.  Community service delivery centres | Home visits  Parenting support  Family support  Childcare services  Early learning programs  Early development of social and communication skills  Services for abused children  Child nutrition programs  Community events  Mass education programs  Community healthcare/development |
| **Program** | **Target Group/s** | **Partnering agencies** | **Highlights of Program model** | **Activities** |
| ***Every Chance for Every Child***  (2003 – 2007, South Australia)  [38] | All children  (0-8 years) | Dept of Human Services  Dept of Education & Children’s Services | Based on key action areas:  Providing more effective support for families.  Strengthening early childhood development and learning.  Strengthening the capacity of communities to be more supportive of families.  Providing more effective and better coordinated programs and services for children and families. | Home visits  Parenting support  Family support  Early learning programs  Antenatal support |
| ***Every Chance for Every Child*** (Victoria)  2008 - 2011 [39] | Vulnerable children, young people and families | Human services depts.  Legal services  Children’s Guardian | (Details not available) | Attending children needs  Child abuse and neglect  Services for out of homecare children  Community-based child and family services |
| ***Best Start*** (Victoria)  2002 [40] | Disadvantaged children (from pregnancy to eight years of age), families and communities | Local bodies/councils  Families  Community partners  Health services  Education services | Working in partnerships: specific focus on improving service co-operation and co-ordination.  Utilization of existing and new service infrastructure. | Home visits  Parenting support  Family support  Early learning programs  Education and schooling  Breastfeeding support  Child nutrition programs  Community healthcare/development |
