# Supplementary material for: Child Health Partnerships: a review of program characteristics, outcomes and their relationship
Source: BMC Health Serv Res. 2010 Jun 17;10:172. doi: 10.1186/1472-6963-10-172 (PMC2908613; doi:10.1186/1472-6963-10-172)
Supplement: Additional file 2 — Child Health Partnerships Projects -Evaluation designs and outcomes [41-46] [file 1472-6963-10-172-S2.DOC]

**Child Health Partnerships Projects -Evaluation designs and outcomes**

| **Program** | **Evaluation design** | **Evaluation outcomes** |
| --- | --- | --- |
| ***Sure Start: Local Programs (SSLP)***  (1999-2005, UK) [14] | **Quasi-experimental cross-sectional study** (intention to treat design) [27]  Program communities were compared with “Sure Start to be” communities [41]  **Four components**:  National survey of selected Program Rounds conducted annually over 2-3 years  Case studies of 10% of the programs in the survey sample  Thematic studies  Cost-effectiveness analysis [42]  **Aspects of partnerships evaluated**: management and coordination; access for families to local program provision and services; community involvement; allocation of resources; the quantity and quality of services provided by local programs [43] | **Partnership formation:** Successful partnership formation among different stakeholders at local level - areas with a previous history of joint work progressed relatively rapidly. Time-scale for optimum program development was longer than expected.  **Service delivery:** Improved service provision/access - diverse pattern of effort and provision of access to the program. Supporting parents and families responded satisfactorily. More vulnerable children (eg. special needs, child protection) identified in program areas [41]. Better service utilization by parents and families - most disadvantaged families performed less well in relation to somewhat less disadvantaged families. SSLPs led by health services reported better outcomes. [27]  **Child and parenting outcomes:** Improved parenting outcomes - high parental involvement; mother’s care for children warmer and more acceptable manner; low level of father involvement; modest influence on parenting and family behaviour.  **Economic evaluation:** Fully-functioning programs established by 3 years; most deprived areas spend more per child than other Sure Start areas; one sixth of expenditure was on play, learning and childcare; each one seventh of expenditure was on healthcare, outreach and home visiting, and support for parents; local programs were slow in delivering capital programs; half of local programmes had only spent 15% capital allocations. Health-led programs utilized money more quickly [42]. |
| ***Sure Start: Children Centres*** **(*Every Child Matters)***  (2005 UK)  [31] | **Quasi experimental observational** study comparing enrolled children and families with similar families in England [24].  Data collection conducted two years apart by different research teams. | **Partnership formation:** Already established in Sure Start Local Programs  **Service delivery:** Improved service provision/access; mothers receiving more services  Better service utilization by parents and families - use of more services designed to support child and family development  **Child and parenting outcomes:** Positive early childhood development in major domains - Better social development, exhibiting more positive social behaviour and greater independence/self-regulation in children; no significant effect on child cognitive and language development; improved parenting outcomes –low problematic parenting, higher level of home learning environment; positive social outcomes of children resulted from better parenting; no significant effect on maternal wellbeing or father involvement in parenting; improved immunization rates; less accidental injuries [24, 28] |
| ***Early Head Start***  (1994 California USA)  [15] | **Randomized trial** with Early Head Start families and control group.  Impact assessment based on 3 program modes on: Child development measures;  Parenting and home environment measures;  and  Measures of parent health and mental health, family functioning, and Self-Sufficiency. | **Partnership formation:** Not reported  **Service delivery:** Strong pattern of impacts among mixed-approach programs (both home and centre-based services)  **Child and parenting outcomes:** Positive early childhood development in major domains - cognitive development, language development and social-emotional development; Improved parenting outcomes -significant favourable impacts on a wide range of parenting outcomes - fathering and father-child interactions.  **Economic evaluation:** These improvements occurred despite the fact that average family income did not increase significantly.[15] |
| **Program** | **Evaluation design** | **Evaluation outcomes** |
| ***Toronto First Duty*** (2002, Canada).  [26, 34, 35] | **Quasi-experimental study**  In-depth analysis of case studies  **Indicators of Change** - Benchmark scale (1-5) of continuum of partnership arrangements provided with program indicators in key elements (local governance, seamless access, early learning environments, early childhood staff team, and parent participation) [44]  **Early Development Instrument** (EDI) to assess child health outcomes | **Partnership formation:** Successful partnership formation among different stakeholders at local level; Effective service cooperation and coordination in partnerships - Partnerships matured along service cooperation and coordination along a continuum of partnership arrangements; Integration improved the quality of early child health programs.  **Service delivery:** Program hours and the participants increased at all of the sites -indicating Improved service provision / access and Better service utilization by parents and families  **Child and parenting outcomes:** Improved parental engagement and parenting outcomes; positive early childhood development in major domains - social; emotional; and language. Changes were not seen in physical development or communication and general knowledge; improved nutrition and eating habits of children [26]  **Economic evaluation:** The costs of the integrated and traditional program are same. ie. Cost-effective. |
| ***Families First*** (New South Wales)  1998 - 2002 [36] | **Process evaluation using triangulated methodology** -multiple qualitative and quantitative data collection techniques  **Area reviews** to assess early, intermediate and established phases  **Methods**: documents reviews, observation studies, interviews, focus groups and surveys; assessment of service integration [6] [37]  **Outcome indicators** for children families & communities [45] | **Partnership formation:** Successful partnership formation among different stakeholders at local level - Significant gains in developing structures and processes  Effective service cooperation and coordination in partnerships –a coordinated service network system established. Fostered relationships between service providers.  Restriction of participation to government agencies.  **Service delivery:** Improved service provision / access - more families reached, particularly those that are harder to reach.  **Child and parenting outcomes:** Not reported.  Limited effects on eg. Housing, schooling.  **Economic evaluation:** Not done. |
| ***Stronger Families and Communities*** (Common Wealth)  2004-2008 [13] | A wide range of quantitative and qualitative methodologies was used by different programs to report outcomes including imposing limitations in data quality  **Process evaluation**: service mapping, service coordination study, partnership model study  **Outcome evaluation**: longitudinal survey of families in 10 sites and monitoring secondary data  **Cost-effectiveness study**  **Themed studies**  **Practice profiles** | **Partnership formation;** Successful partnership formation among different stakeholders at local level -76% programs developed partnerships between services; effective service cooperation and coordination in partnerships - 75% of programs - ‘Outstanding’ or ‘Generally Successful’; education, general child and family services contributed to service integration. Existence of supportive networks for sites. Moderate levels of day-to-day coordination and high levels of effective partnerships at baseline; wider community involvement - Greater than expected interest and commitment from a larger community.  **Child and parenting outcomes:** Improved parenting outcomes  Initial improved participation and enhanced trust among partners lead to improved family and community wellbeing as the end result.  **Economic evaluation:**  Both short-term and long–term cost-benefits achieved by the majority of programs. Two thirds of programs are likely to sustain even after funding ended. [46] |
| ***Best Start*** (2002, Victoria) [40] | **Quasi-experimental study** with several control/comparator groups: Best Start versus non-Best Start areas, and pre- and post-Best Start analyses.  **Mixed methods** based on Owen’s typology for Development, Clarification, Improvement, Monitoring and Impact:  **Measures** Process indicators, site evaluation profiles, VicHealth Partnership Analysis Tool, Service Co-operation and Co-ordination Survey Tool, Omnibus Survey of Parents, Vulnerable Families strategy and site visits/interviews | **Partnership formation:** Successful partnership formation among different stakeholders at local level - Partnerships improved in all dimensions over time. Effective service cooperation and coordination in partnerships. Wider community involvement  **Service delivery:** Service provision / access - Improved inclusiveness of primary level services and no improvement in access. More vulnerable families engaged. Access to maternal and child health programs significantly improved. Better service utilization by parents and families  **Child and parenting outcomes:** Improvement in breastfeeding and maternal and child health visiting indicators; No significant changes in parenting confidence. No change in community/local safety.  **Economic evaluation:** Not done. |
